# One-year Increment Staging Incidence of Esophageal Adenocarcinoma With Enhanced Ethnicity Surveillance, Epidemiology, and End Results Program 18 Sampling, 2000–2017

**DOI:** 10.1016/j.gastha.2022.08.009

**Published:** 2022-08-28

**Authors:** E.M. Montminy, M. Zhou, J.H. Bommireddipally, J.J. Karlitz, S. Wani

**Affiliations:** 1 Division of Gastroenterology, Tulane University School of Medicine, New Orleans, Louisiana; 2 Southborough, Massachusetts; 3 Department of Internal Medicine, Tulane University School of Medicine, New Orleans, Louisiana; 4 Division of Gastroenterology and Hepatology, University of Colorado Anschutz Medical Center, Aurora, Colorado; 5 Division of Gastroenterology and Hepatology, Denver Health Medical Center, Denver, Colorado

Esophageal adenocarcinoma (EAC) incidence rates (IRs) are rising in the United States.^1^ Additionally, a recent study utilizing the Surveillance, Epidemiology, and End Results Program 9 (SEER 9, sampling approximately 9% of the US population) demonstrated most patients with EAC present with advanced stage (analyzed as regional/distant combined).^1^ With rising EAC IRs and increasing EAC risk factor prevalence across all ethnicities, a more granular and comprehensive assessment of EAC staging by age and ethnicity is required as this could impact public health and current screening guidelines for Barrett’s esophagus (BE), the only identifiable premalignant condition for EAC. Current guidelines suggest screening with endoscopy in individuals with chronic gastroesophageal reflux disease with the presence of other risk factors (male, age >50 years, white race, smoking, obesity, and family history of BE/EAC).^2^ While white race has been predominantly utilized for profiling EAC risk demographics, this race-based recommendation was recently questioned in a document published by the American Gastroenterological Association.^3^ The primary aim of this study was to utilize the SEER 18 database to assess ethnicity and stage-stratified trends in EAC IRs and determine differences in distant-stage IRs. SEER 18 samples approximately 28% of the US population and has improved ethnicity sampling compared to SEER 9 due to additional urban registries. Compared to prior studies in which age blocks (40–49, 50–59 etc.) were analyzed, we seek to analyze IR changes in 1-year age increments over the 2000–2017 SEER 18 data set. This type of detailed analysis can allow assessment of changes in EAC staging distributions as patients approach index screening age recommendations and thereafter.^4^ Additionally, we aimed to utilize rate-ratio analysis to compare distant to localized EAC IRs to gauge stage severity as age progresses.

Using the 2000–2017 SEER 18 data set, mean 1-year interval age-adjusted EAC IRs per 100,000 person-years (PY) were obtained for ages 40–70 years using SEER∗Stat (version 8.3.9.1). IRs were adjusted to the year 2000 US population. SEER 18 was chosen given higher-quality data sets and inclusion of more urban/diverse patient populations than other SEER registries.^5^ EAC IRs were stratified by stage (in situ, localized, regional, and distant) and ethnicity (all ethnicities combined, non-Hispanic white, non-Hispanic black, and Hispanic). Rate-ratios were calculated to determine if each age’s distant EAC IR was significantly higher than corresponding localized EAC IRs. Confidence intervals were generated by SEER∗Stat with the recommended Tiwari 95% confidence interval option to determine significance. Rate-ratios were considered significant if confidence intervals did not cross 1. Joinpoint Regression Program (National Cancer Institute, version 4.8.0.1) was used to quantify all ethnicities/stages combined EAC IR annual percent change (APC) trends. For APCs, Monte Carlo permutation method was utilized with standard errors from SEER∗Stat to determine significance.

A total of 23,386 EAC cases were reported over 2000–2017. Regarding all ethnicities combined (Figure A), distant EAC had the highest IR for every age except for age 70 when regional IR was higher (regional 4.2/100,000 PY vs distant 3.9/100,000 PY). Furthermore, distant EAC IRs remained significantly higher than localized IRs at every age. Among subgroups, non-Hispanic white EAC IRs (Figure B) represented the highest peak distant-stage IR (age 67–69, 5.9/100,000 PY). Non-Hispanic white distant-stage IR was higher than non-Hispanic black (Figure C) and Hispanic IRs (Figure D) at each corresponding age except for age 40 (0.2/100,000 PY). Non-Hispanic white and Hispanic distant IRs were significantly higher than localized IRs at nearly all corresponding ages. Non-Hispanic black IRs displayed numerically predominantly distant-stage EAC as age progressed although confidence intervals were wide. Regarding APC in all ethnicities/all stages combined (Figure A1), APC increased the fastest from age 40 to 49 (19.7%, *P* < .05). From age 49 to 61, APC significantly increased, although at a lesser rate, 11.4% (*P* < .05), and less so from age 61 to 70 years (5.4%, *P* < .05).

**Figure:**
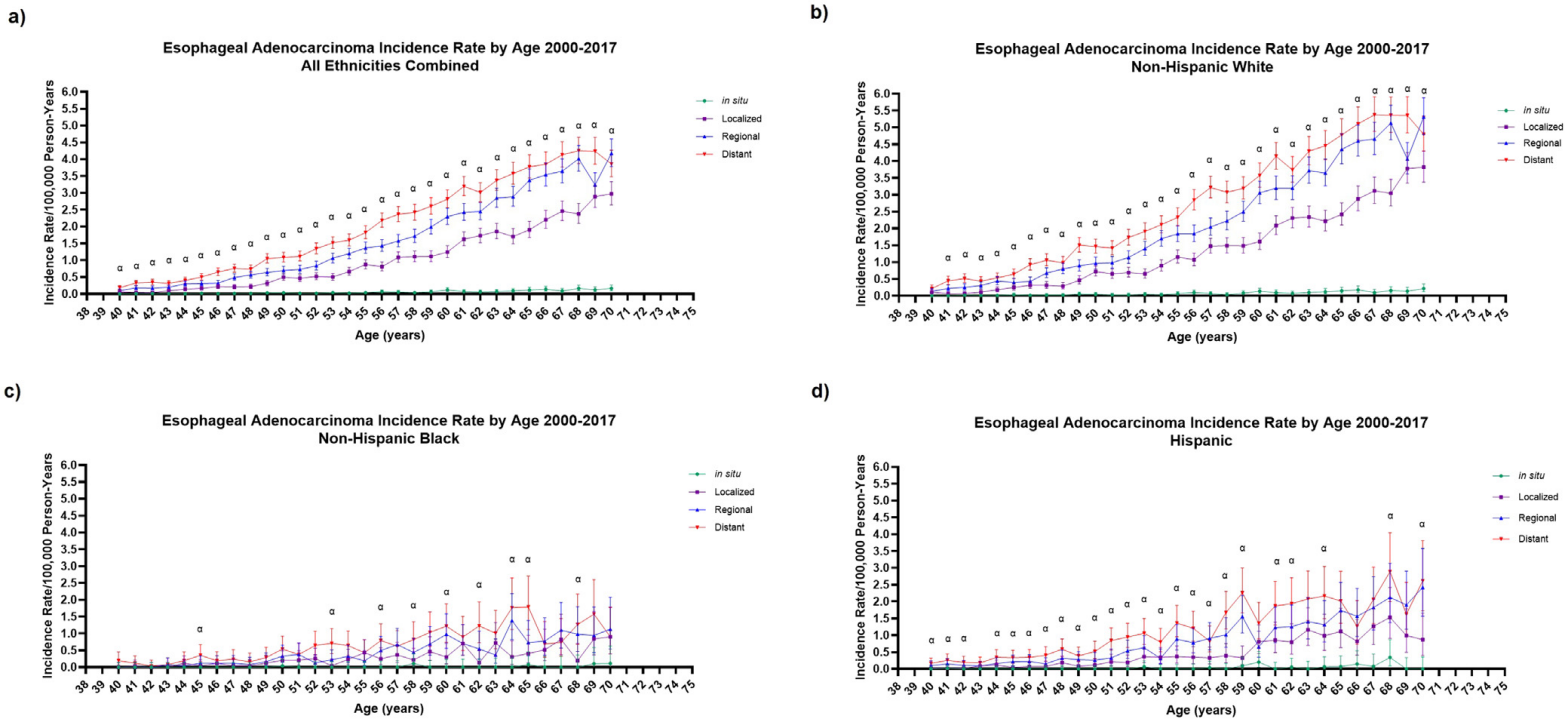
SEER 18 1-year staging incidence in age 40–70 in (A) all ethnicities combined, (B) non-Hispanic white, (C) non-Hispanic black, and (D) Hispanic patients. Stages include distant (red), regional (blue), localized (purple), and in situ (green). Alpha indicates rate ratio of distant IR is significantly higher than localized IR with *P* < .05.

The results of this study demonstrate predominant distant-stage EAC as age progresses from 40 to 70 years over the 2000–2017 time period except in non-Hispanic black patients. Our findings are unique in that they are the first to report EAC stage analysis in 1-year intervals utilizing the SEER 18 expanded racial/ethnic sampling. Our analysis also shows distant EAC is significantly increased compared to localized disease starting prior to age 50 (the typical index screening age for BE), particularly in all ethnicities combined and non-Hispanic white subgroups. Because distant EAC predominates prior to age 50 and persists thereafter in most subgroups, current screening recommendations and efforts to identify at-risk patients are potentially missing early-stage EAC. Predominant distant EAC throughout our entire population and substantially rising APCs as index screening approaches (although low overall absolute IRs) suggest that a renewed effort similar to recent colorectal cancer initiatives may be necessary for earlier detection.^6^ In addition, the substantial increase in APC values even among EAC patients older than 50 years suggests failure in identification of at-risk patients through current screening strategies.

With expanding US Hispanic populations and predominantly distant disease in this subgroup, improved outreach efforts are needed to identify at-risk Hispanic individuals for BE/EAC through endoscopic or noninvasive screening modalities. Unlike other published guideline statements, the 2019 American Society for Gastrointestinal Endoscopy Barrett’s esophagus screening guidelines do not reference white race when profiling BE/EAC risk and represents a paradigm shift in EAC risk stratification.^3^^,^^7^ Given Hispanic ethnic heterogeneity and 20% of Hispanic patients identifying as white, Hispanic heritage impact on EAC risk remains unclear with epidemiologic data alone.^8^ Hence, this guideline change alone should not be considered as endorsement of universally screening Hispanic populations for now.

Limitations include a case-controlled analysis and inability to characterize nomenclature bias impacts on in situ stage distribution. Regarding low in situ IRs throughout all subgroups, SEER registries only report an in situ case if labeled as “carcinoma in situ” (outdated term) and does not count cases labeled high-grade dysplasia. We suspect a degree of nomenclature bias is present, but confirmatory studies are required. Currently, national cancer organizations are putting forth recommendations to address this bias.^9^

With more evidence of predominantly distant-stage EAC across all ages and ethnicities, future efforts need to refine the current paradigm of BE screening to improve population-level EAC outcomes.

## Supplementary Materials

Supplemental Caption

Figure A1
