# Supplementary material for: One-year Increment Staging Incidence of Esophageal Adenocarcinoma With Enhanced Ethnicity Surveillance, Epidemiology, and End Results Program 18 Sampling, 2000–2017
Source: Gastro Hep Adv. 2022 Aug 28;2(1):5–7. doi: 10.1016/j.gastha.2022.08.009 (PMC11307698; doi:10.1016/j.gastha.2022.08.009)
Supplement: Supplemental Caption [file mmc1.docx]

**Supplemental Figure Legend for 1-year Staging Incidence of Esophageal Adenocarcinoma with Enhanced Ethnicity SEER 18 Sampling, 2000-2017**

**Supplemental Figure 1. SEER 18 Distant EAC IR Annual Percent Change in Age 40-50 (blue), 50-61 (green), 61-70 (red), All Ethnicities/Stages Combined.** *indicates significantly elevated APC to p<0.05.
