# Supplementary figures and images for: One-year Increment Staging Incidence of Esophageal Adenocarcinoma With Enhanced Ethnicity Surveillance, Epidemiology, and End Results Program 18 Sampling, 2000–2017
Source: Gastro Hep Adv. 2022 Aug 28;2(1):5–7. doi: 10.1016/j.gastha.2022.08.009 (PMC11307698; doi:10.1016/j.gastha.2022.08.009)

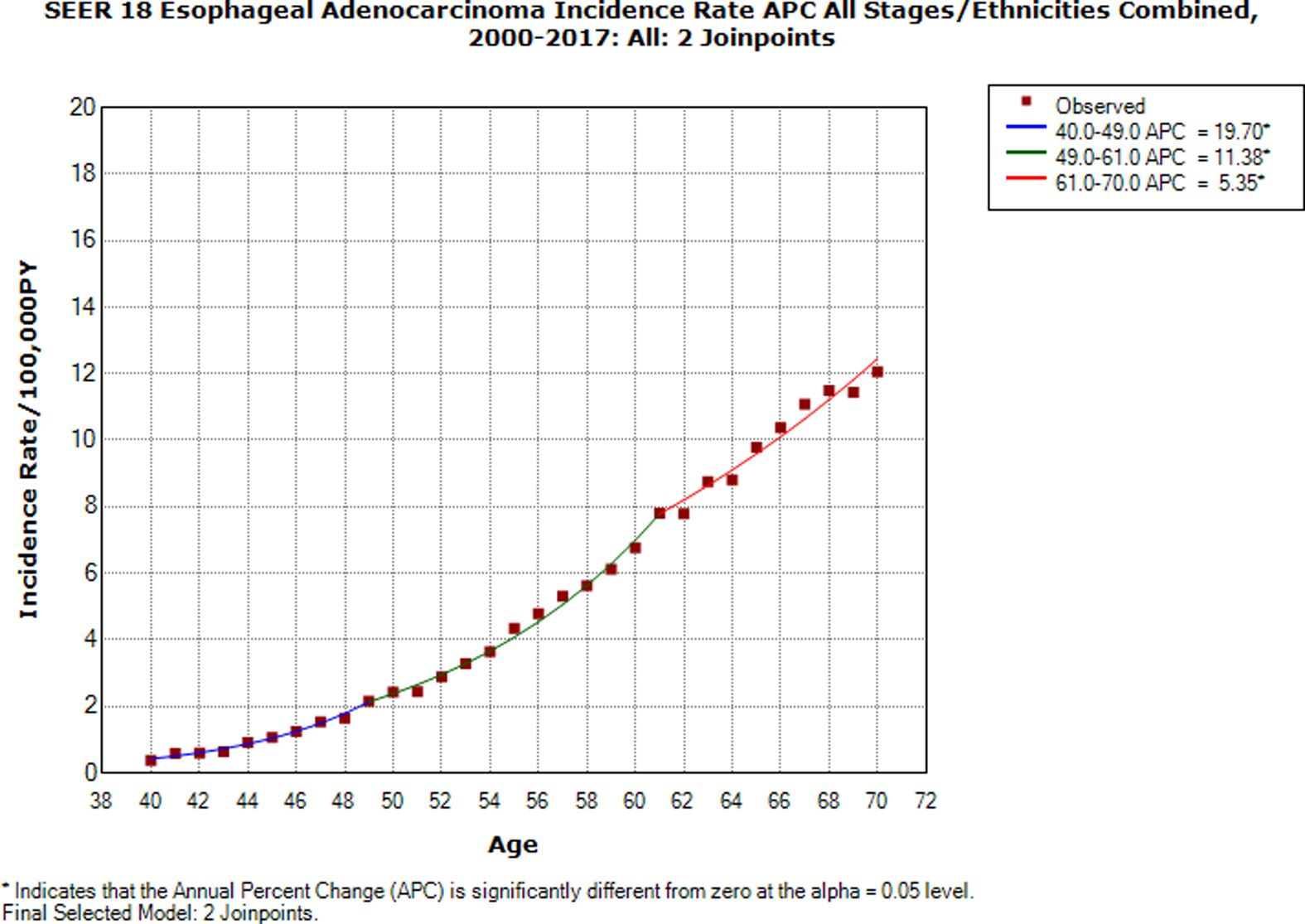

Supplement: Figure A1 [file figs1.jpg]
